# Quiescent Human Mesenchymal Stem Cells Are More Resistant to Heat Stress than Cycling Cells

**DOI:** 10.1155/2018/3753547

**Published:** 2018-12-24

**Authors:** L. L. Alekseenko, M. A. Shilina, O. G. Lyublinskaya, J. S. Kornienko, O. V. Anatskaya, A. E. Vinogradov, T. M. Grinchuk, I. I. Fridlyanskaya, N. N. Nikolsky

**Affiliations:** ^1^Institute of Cytology, Russian Academy of Sciences, Saint Petersburg, Russia; ^2^Peter the Great St. Petersburg Polytechnic University, Saint Petersburg, Russia

## Abstract

Quiescence is the prevailing state of many cell types under homeostatic conditions. Yet, surprisingly, little is known about how quiescent cells respond to environmental challenges. The aim of the present study is to compare stress responses of cycling and quiescent mesenchymal stem cells (MSC). Human endometrial mesenchymal cells (eMSС) were employed as adult stem cells. eMSC quiescence was modeled by serum starvation. Sublethal heat shock (HS) was used as a stress factor. Both quiescent and cycling cells were heated at 45°C for 30 min and then returned to standard culture conditions for their recovery. HS response was monitored by DNA damage response, stress-induced premature senescence (SIPS), cell proliferation activity, and oxidative metabolism. It has been found that quiescent cells repair DNA more rapidly, resume proliferation, and undergo SIPS less than proliferating cells. HS-enforced ROS production in heated cycling cells was accompanied with increased expression of genes regulating redox-active proteins. Quiescent cells exposed to HS did not intensify the ROS production, and genes involved in antioxidant defense were mostly silent. Altogether, the results have shown that quiescent cells are more resistant to heat stress than cycling cells. Next-generation sequencing (NGS) demonstrates that HS-survived cells retain differentiation capacity and do not exhibit signs of spontaneous transformation.

## 1. Introduction

Human MSC as promising cell therapy candidates are under intensive investigation. Their differentiation abilities, immunomodulatory effects, and homing properties offer potential for augmenting regenerative capacity of many tissues. Mesenchymal stem cells are fibroblast-like adherent cells, which can be isolated from various tissues, such as bone marrow, umbilical cord, adipose tissue, peripheral blood, spleen, and skin [1]. Currently, MSC derived from endometrium (eMSC) attract growing attention. Comparing with other MSC types, eMSC show a higher vasculogenic, anti-inflammatory, and immunomodulation potential [2, 3]. These valuable features are associated with a special role of eMSC in endometrial regrowth every month. Cultured eMSC are applied in clinical trials and encouraging results have been reported [4, 5].

A major impediment to the development of MSC-based therapies, however, is poor cell survival at the site of injury. Generally, the harsh environment of injured tissue is associated with oxidative stress, chronic inflammation, fibrosis, extracellular matrix degradation, and immune rejection [6]. This is why the stress response of cultivated human stem cells is under intensive study [7–11].

Cells exposed to stress may respond differently: undergo differentiation, senescence (SIPS), apoptosis, or necrosis. The choice depends on the cell type and stress strength. Mild stress may improve differentiation of stem cells [12, 13]. The outcome for unbearable stress is necrosis. Sublethal doses of various stressors mostly produce senescence (SIPS) and sometimes later apoptosis.

Heat stress (heat shock, hyperthermia) is one of the well-studied types of stress. It can affect a variety of cell types. Hyperthermia can accompany therapeutic procedures, such as stem cell-based therapy and cancer treatment. Hyperthermia changes the blood circulation and oxygen supply reduces the ATP level and increases anaerobic metabolites and activity of DNA repair proteins. It has various effects on the immune system, such as increased peripheral blood mononuclear cell proliferation, increased cytotoxic activity of CD8^+^ T cells and augmented secretion of IFN-*γ* by these cells. It also causes the secretion of inflammatory cytokines, such as TNF-*α* and IL-1, alters the migration of Langerhans cells, and provokes lymphocyte homing into secondary lymphoid tissues. Heat-shocked MSC can inhibit tumor growth and enhance tumor cell death [14]. Hyperthermia was applied in vivo to stimulate osteogenesis [15, 16]. It was demonstrated that mild heat stress promoted myoblast differentiation [17] and osteogenesis of bone marrow MSC [18, 19]. Severe HS common for orthopedic procedures induced apoptosis and necrosis in cultured osteoblasts [20, 21]. Proliferation of dental follicle stem cells was stimulated by increased temperature [22, 23]. Enlarged temperature enhanced the proliferation of UCV-MSC cocultured with mononuclear cells of the peripheral blood as well as expression of IL-10, TGF-*β*1, and FOXP3 mRNAs. It had no effect on IL-17A and IFN-*γ* secretion and reduced CXCL12 [24]. In our experiments, sublethal temperature has induced preliminary senescence [25] which is a mechanism of maintenance of MSC genetic stability by excluding damaged cells from the proliferation pool.

In a living body, stem cells may long reside in the dormant state entering the cell cycle in response to local signals of damage and other regeneration needs. Quiescence is the prevailing state of many cell types under homeostatic conditions. Proliferating cells in culture can be induced into quiescence by mitogen withdrawal under serum deprivation [26]. Serum deprivation (SD) for 48 hours shifted MSC into a quiescent state in which cells remained metabolically healthy but nonproliferative with reduced levels of RNA and protein synthesis. Upon reintroduction to standard culture conditions, SD-MSC restored proliferation and properties of parental cells. Quiescence preconditioning-afforded MSC increased viability under low oxygen or total glucose depletion [27]. Yet, surprisingly, little is known about how quiescent cells respond to environmental challenges. In this connection, the aim of the present study is to compare heat stress (HS) responses of cycling and quiescent eMSC. Moreover, we examined HS-survived and HS-expanded cells for their differentiation potency and tumorigenic risk using next-generation sequencing (NGS).

## 2. Methods

### 2.1. Cells

We used MSC isolated from desquamated endometrium of menstrual blood (eMSC) of healthy donors [28]. The cells were cultured in DMEM/F12 (Gibco, UK) with 10% fetal bovine serum (HyClone, USA), 1% GlutaMAX (Life Technologies, Japan), and 1% penicillin-streptomycin (Gibco, UK) in plastic 30 mm Petri dishes (Nunc, Denmark) at 37°C, 5% CO_2_, and 95% humidity. For microscopic experiments, cells were grown on glass coverslips. Cells from the 5–7th passages were used for experiments.

For accumulation of quiescent cells, eMSC that reached the monolayer were harvested using 0.05% trypsin-EDTA solution (Gibco, UK) and plated at a density of 10 × 10^3^ cells/cm^2^. The next day, the medium was changed for serum-free medium for 30 h.

### 2.2. Cell Heating of Quiescent and Proliferating Cultures

Before HS, quiescent cells were added with serum for 2 h to heat quiescent and proliferating cells under equal conditions. Both quiescent and proliferating cells were exposed to sublethal HS at 45°C for 30 min [29] in parafilm-covered plates in water bath. Previously, we demonstrated that these conditions are sublethal for cycling MSC and a part of the cells is able to survive [25]. Cells of both types exposed to HS were returned under 37°C for recovery in serum-containing growth medium for 72 h.

### 2.3. Immunofluorescence

eMSC grown on coverslips were fixed with 4% formalin in phosphate-buffered saline (PBS) for 15 min, permeabilized with 0.1% Triton X-100, blocked with 1% bovine serum albumin for 30 min, treated with primary antibodies for 45 min, washed with 0.1% Tween 20, treated with secondary antibodies for 45 min, washed with/0.1% Tween 20, and counterstained with 1 *μ*g/mL DAPI. Then the coverslips were mounted on ProLong Gold antifade reagent (Thermo Fisher Scientific, USA). The primary antibodies used were mouse monoclonals against Hsp70 [30], *γ*H2AX (Abcam, USA), and rabbit polyclonal against Ki-67 (Abcam, USA). Goat anti-mouse IgGAlexa 488 (Invitrogen, USA) and goat anti-rabbit DyLight 567 (Invitrogen, USA) were applied as secondary antibodies. Images were captured using Leica TCS SP5 confocal microscope (Leica Microsystems, Germany) equipped with solid-state lasers for excitation (405, 488, and 543 nm) and HCX PL APO CS 40x oil immersion objective (NA=1.3, Leica). Adobe Photoshop software was used to view and acquire images.

### 2.4. Western Blot Analysis

The cells lysed by incubation in RIPA Buffer (50 mM Tris HCl, 150 mM NaCl, 1 mM EDTA, 1 mM EGTA, 10% glycerol, 1% Triton X100, 1 mM Na3VO4, 1 mM NaF and 0.5 mM PMSF) and cocktail of protease inhibitors (1 : 500, Sigma, United States)) for 15 minutes on ice were scrapped off from the plates and centrifuged for 20 minutes at 15000 g. The proteins were denatured by addition of electrophoretic buffer (40 mM Tris (pH 6.8), 10% SDS, 20% 2-mercaptoethanol and 40% glycerol) to the supernatant and further incubation at 100°C for 5 minutes. Protein concentration was measured by Bradford's method using ovalbumin for construction of the calibration curve. Proteins were separated electrophoretically in 10% polyacrylamide gel with subsequent transfer onto a HybondC extra nitrocellulose membrane (Amersham Pharmacia Biotech, Sweden). For detection of protein bands, the following primary and secondary antibodies (all from Cell Signaling) were used: anti-phospho-pRb (Ser807/811, 8516S), anti-cyclinA2 (4656S), anti-GAPDH (2118S), GAR-HRP (7074S), and GAM-HRP (7076S). The peroxidase activity of GAR-HRP and GAM-HRP conjugates was detected by enhanced chemiluminescence (ECL) reaction (Amersham, Sweden). Chemiluminescent signal was recorded by exposure to an X-film from CEA RP NEW (CEA AB, Sweden). For densitometric analysis of protein bands, ImageJ software was used.

### 2.5. Detection of ROS

For the measurements of intracellular ROS level, fluorescent probe 2′,7′-dichlorodihydrofluorescein diacetate (H2DCFDA, Invitrogen, D-399) was used. H2DCFDA was dissolved in DMSO to obtain a 10 mM stock solution which was further diluted in PBS before use to obtain staining solution. Cells were incubated with 5 *μ*M H2DCFDA staining solution in the dark for 20 min at 37°C, then harvested with 0.05% trypsin-EDTA solution, suspended in a fresh medium and immediately analyzed with CytoFLEX flow cytometer (Beckman Coulter, USA; 488 nm laser). Cells were detected by size and granularity using FSC/SSC dot plot, and cell debris was gated out. Mean fluorescence intensity from 10,000 cells was acquired.

### 2.6. Cell Cycle Analysis

Cells were harvested with trypsin-EDTA solution and suspended in fresh medium. 200 *μ*g/mL saponin (Fluka, NY, USA), 250 *μ*g/mL RNase A (Sigma, St. Louis, MO, USA, R4642), and 50 *μ*g/mL propidium iodide (Sigma, USA) were added to each sample tube. After incubation for 60 min at room temperature, samples were analyzed with CytoFLEX flow cytometer (Beckman Coulter, USA; 488 nm laser). Mean fluorescence intensity from 10,000 cells was acquired. Cell cycle analysis was performed using CytExpert v. 2.0 software (Beckman Coulter, USA).

### 2.7. Viability Analysis

Cells were harvested with trypsin-EDTA solution and suspended in fresh medium. 50 *μ*g/mL propidium iodide (Sigma, USA) was added to each sample tube. After incubation for 5 min at room temperature, samples were analyzed with CytoFLEX flow cytometer (Beckman Coulter, USA; 488 nm laser). Mean fluorescence intensity from 10,000 cells was acquired. Cells were gated by size and granularity using FSC/SSC dot plot, and cell debris was excluded from the analysis.

### 2.8. RT-PCR and qRT-PCR Assays

To analyze gene expression, total RNA was isolated with RNeasy Micro Kit (QIAGEN) according to the manufacturer's instructions. RNA was quantified in the NanoDrop ND-1000 Spectrophotometer (NanoDrop Technologies Inc., Wilmington, DE, USA). cDNA was obtained by reverse transcription of 500 ng RNA using the RevertAid H Minus First Strand cDNA Synthesis Kit (Fermentas) according to the manufacturer's instructions. It was subsequently amplified with specific primers, using DreamTaq™ PCR Master Mix (2X) (Thermo Fisher Scientific) with CycloTemp amplificator. The electrophoresis of amplified products was performed in 2% agarose gel with TAE buffer and ethidium bromide. 100 kb DNA ladder (Fermentas, Lithuania) was used as molecular weight markers. Amplified products were visualized in UV light (302 nm) with a transilluminator and registered with a digital Canon camera. For qRT-PCR, cDNA was amplified with specific primers, using EvaGreen® dye (Biotium) and DreamTaq™ PCR Master Mix (2X) (Thermo Fisher Scientific) in the Bio-Rad CFX-96 real-time system (Bio-Rad, CA, USA), according to the kit's enclosed protocol. The volume of RT and PCR reactions was 20 *μ*L. Expression of target genes was normalized to GAPDH or actin gene. Primers and reaction conditions are presented in Table 1. All amplification reactions were performed in triplicates. Experiments were repeated at least three times.

### 2.9. SA-*β*-Gal Activity Assay

Cells expressing senescent-associated *β*-galactosidase (SA-*β*-Gal) were detected with senescence *β*-galactosidase staining kit (Cell Signaling Technology) according to manufacturer's instructions and quantified microscopically by counting X-Gal-positive cells among not less 500 cells in random fields of view.

### 2.10. Next-Generation Sequencing (NGS)

Cells that survived HS were subcultured for 6 passages and subjected to NGS. NGS was done by parallel measurement of three biological samples: unheated cells and cells heated in quiescent and proliferative stages. Sample preparation for NGS and sequencing on the Illumina platform were performed in «Genotek» company (Moscow, Russia). RNA was extracted using PureLink RNA Mini Kit (Ambion, Life Technologies). cDNA libraries were prepared using NEBNext® mRNA Library Prep Reagent Set for Illumina® (New England Biolabs). Quality control of prepared libraries was made using Bioanalyzer 2100 (Agilent Technologies). Sequencing of cDNA libraries was done on HiSeq 2500 (Illumina) in Rapid Run Mode with a read length of 100 nt.

The differential gene expression and gene modules enriched in differentially expressed genes were done similarly to previous works [31]. The biological processes were taken from GO database. NCBI BioSystems was used as a source of molecular pathways.

To obtain GO biological processes and NCBI BioSystems pathways related to differentiation, we searched for gene modules containing in their titles terms related to various types of mesenchymal stem cell differentiation. We selected gene modules containing in titles terms “differentiation”, “osteo”, “adipo”, “neuro”, “BDNF”, “oligodendro”, “chondro” with significance level *p* < 0.005 and *q* < 0.15. The data on key transcription factors (TFs) regulating mesenchymal stem cell differentiation was taken from [32].

To evaluate cell transformation potential, we used the gene set provided by [33]. The authors analyzed DNA sequence of more than 8,200 tumor-normal pairs and identified 49 oncogenes (ONGs) and 50 tumor suppressors (TSGs) playing the most important role in cancer initiation, progression, prevention, and suppression.

### 2.11. Statistical Analysis

Experiments were performed in triplicate. The results are expressed as mean ± SD. Student's *t*-test was used to determine the statistical significance of differences between two groups; one-way ANOVA with post hoc Tukey HSD test was used to determine the significance of differences among groups. The null hypothesis was rejected at the 0.05 level of significance.

## 3. Results

### 3.1. eMSC Accumulation in the Quiescent State

**Figure 1:**
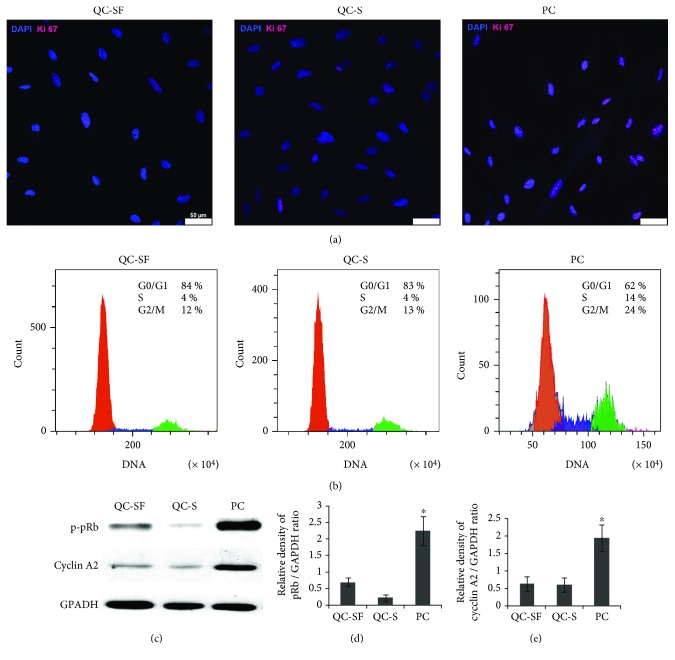
Accumulation of quiescent (Go/G1) cells by serum starvation. (a) Immunofluorescence assay of proliferation of quiescent serum-free cells (QC-SF), quiescent cells in 2 h after serum addition (QC-S), and proliferating cells (PC) with anti-Ki-67 antibodies. Unlike PC, only single QC-SF and QC-S are Ki-67 positive. (b) FACS assay of cell cycle distribution of QC-SF, QC-S, and PC. (c) Immunoblot analysis of cyclin A2 and p-pRb levels. (d, e) Densitometric analysis of immunoblots normalized to the GAPDH loading control. Mean ± SD of the three experiments is shown. ^∗^PC difference vs QC-SF and QC-S (*t*-test, ^∗^ *p* < 0.05). Scale bar = 50 *μ*m.

eMSC cultivated in serum-free medium for 30 h stopped proliferation and accumulated in the G0/G1 stage (Figure 1(b)). Most cells became Ki-67 negative (Figure 1(a)). Expression of cyclin A2 and p-pRb proteins common for dividing cells was inhibited (Figure 1(c)). Before HS, quiescent cells were added with serum for 2 h to heat quiescent and proliferating cells under equal cell culture conditions. Quiescent cell cultivation for 2 h in the complete growth medium with 10% serum maintained all characteristics of quiescent cells (Figure 1). Most of these cells were in G0/G1 and Ki-67 negative. A few Ki-67 positive cells in serum-free cultures and at 2 h after serum addition exhibited lower fluorescence intensity compared with cells in proliferating cultures (Figure 1(a)).

### 3.2. HS Induced Expression of Hsp70 in Quiescent and Proliferating eMSC

**Figure 2:**
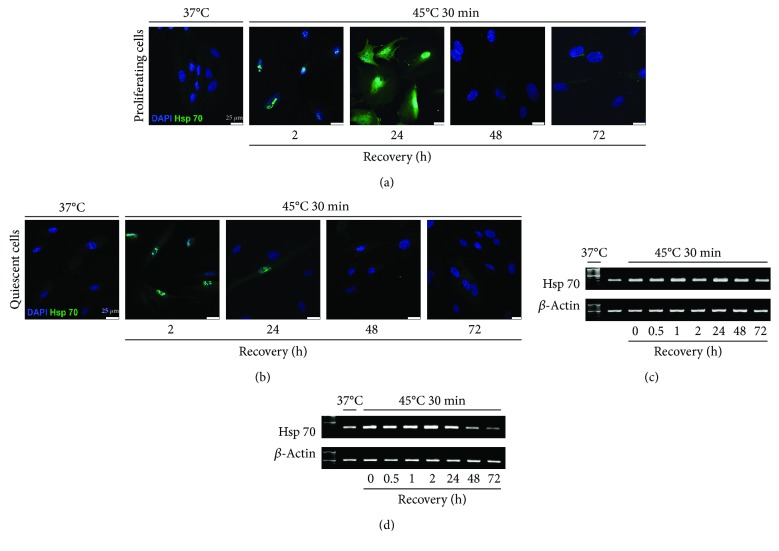
Expression of HSP70 in quiescent and proliferating cells after HS. (a, b) Immunofluorescence assay of (a) proliferating cells and (b) quiescent cells with anti-HSP70 antibodies. Nuclei were contrasted with DAPI; scale bar = 25 *μ*m. (c, d) RT-PCR analysis of the *HSP70* gene level in (c) proliferating and (d) quiescent cells. Data of three independent experiments are presented.

The level of HSP70 is an indication of cell stress response. Measurement of the level and localization of Hsp70 protein showed that heating at 45^o^C for 30 min was stressful for both quiescent and proliferating eMSC.  Figure 2 shows immunofluorescence and PCR assay of HSP70 expression. HS-triggered expression of HSP70 gene was observed after 2 h of recovery in both cell types. It was at the high level in proliferating cells up to 72 h whereas it started to decline in G0/G1-heated cultures at 48 h and dropped close to the level in unheated cells after 72 h. Immunofluorescence assessment showed localization of HSP70 protein in nuclei of both cell types at 2 h after HS. At 24 h after the cell recovery, the pattern of HSP70 distribution was different in proliferating and quiescent cells. HSP70 was virtually not identified in heated quiescent cells but visible in both the nucleus and cytoplasm of heated cycling cells. These findings demonstrate that HS cell response in quiescent and proliferating cultures is different. More rapid return of HSP70 expression after HS to the normal level in quiescent cells during recovery is an indication of their higher resistance to HS.

### 3.3. HS Caused Premature Senescence in Both Proliferating and Quiescent Cultures

**Figure 3:**
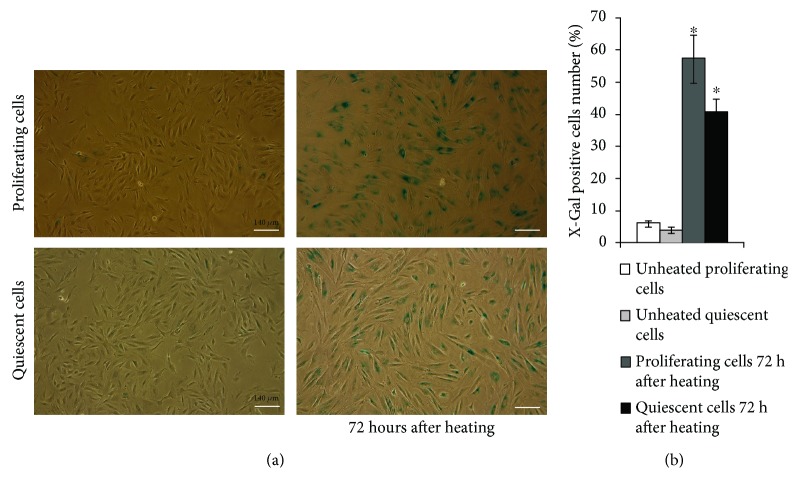
HS induces appearance of SA-*β*-Gal-positive cells in both proliferating and quiescent cultures. (a) SA-*β*-Gal staining. The senescent cells were detected with SA-*β*-Gal staining kit Ob: 10x; scale bar = 140 *μ*m. (b) Quantitative assay of SA-*β*-Gal-positive cells. At least 500 cells from different fields of view were analyzed. Data from three independent experiments are presented. ^∗^The difference vs unheated cells (*t*-test, *p* < 0.05).

The most common MSC stress response is premature senescence [9]. We found previously that eMSC exposed to sublethal HS underwent stress-induced premature senescence (SIPS) [25].  Figure 3 demonstrates that cell number decreases and cells become more flattened and enlarges in cultures of both cell types subjected to HS. Most of these cells are SA-*β*-Gal positive. It is commonly believed that the key features of SIPS in MSC cultures are SA-*β*-Gal staining and modified morphology.

The number of SA-*β*-Gal-positive cells was slightly lower in quiescent than in proliferative cultures exposed to HS.

### 3.4. HS Provokes DNA Damage Response (DDR) in Proliferating and Quiescent eMSC

**Figure 4:**
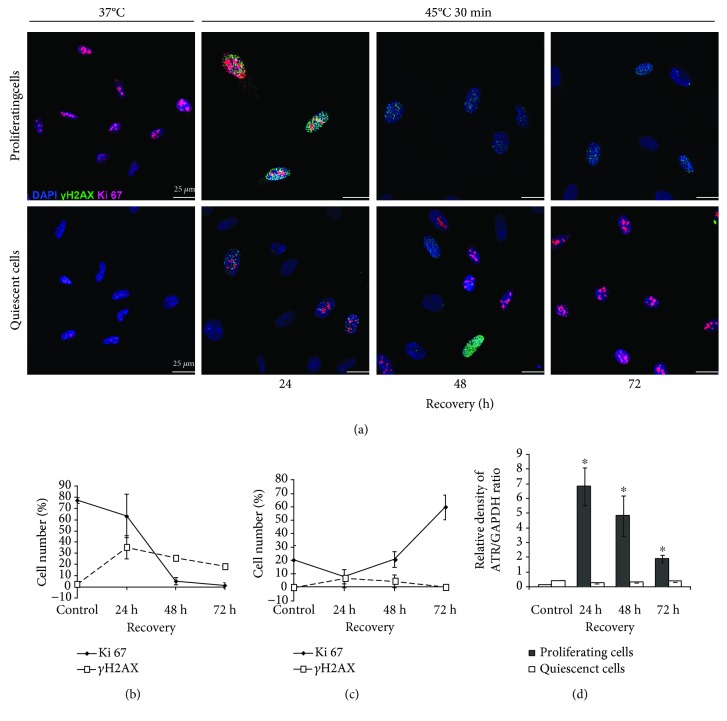
DNA damage response (DDR) of eMSC heated in proliferating and quiescent stages. (a) Immunofluorescence assay of proliferating and quiescent cells with anti-*γ*H2AX and anti-Ki-67 antibodies. Nuclei were contrasted with DAPI; scale bar = 25 *μ*m. (b, c) Quantitation of *γ*H2AX and anti-Ki-67 positive cells in proliferating (b) and quiescence (c) cultures after HS. At least 300 cells from different fields of view were counted. (d) qRT-PCR analysis of the *ATR* level in proliferating and quiescent cells exposed to HS. Mean ± SD of three independent experiments is presented. ^∗^The difference vs unheated cells (*t*-test, *p* < 0.05).

It is commonly believed that SIPS triggering with various stresses is initiated by DNA damage [34, 35]. HS as a stressful factor induces both single- and double-strand DNA breaks. DNA breaks activate DNA damage response. eMSC heating was accompanied with the appearance of *γ*H2AX foci, a DNA damage marker (Figure 4(a)). Maximal focus number was observed at 24 h of recovery, and then it started to decline. However, this tendency differed in heated quiescent and growing cells. After 72 h of recovery, *γ*H2AX foci were visible only in a few cells in cultures heated in quiescence but were identified in many cells heated in the proliferative phase (Figure 4(a)). At this period, cells heated in the quiescent state resumed proliferation and had an increased number of Ki-67-positive cells (Figure 4(c)). Cells that underwent HS in the proliferative state were mostly Ki-67 negative (Figure 4(b)).

In most somatic cells, DDR is accompanied by activation of sensor kinases.  Figure 4(d) shows expression of ATR kinase, a serine/threonine-specific protein kinase involved in sensing of DNA damage. It is seen that ATR is weakly expressed in intact cells of both types. After heating, its expression in cycling cells increased during 24 h and then dropped. In cells heated at the quiescence state, ATR expression was not induced by HS.

### 3.5. Quiescent Cells Exposed to HS Resumed Proliferation Faster than Heated Proliferating Cells

**Figure 5:**
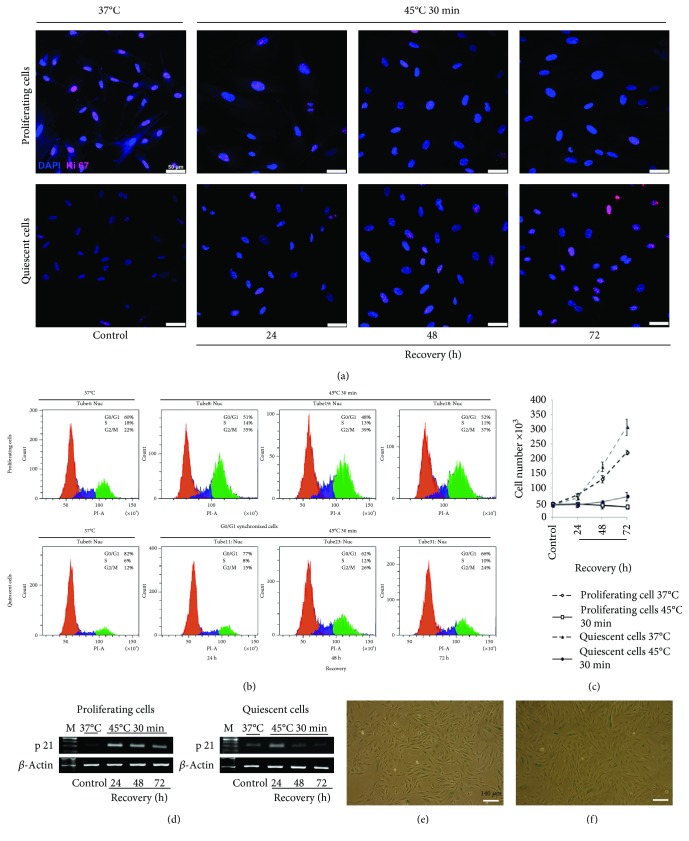
Resumed proliferation of quiescent and proliferating cells after HS. (a) Immunofluorescence assay of proliferating and quiescent eMSC with anti-Ki-67 antibodies. Nuclei were contrasted with DAPI; scale bar = 50 *μ*m. (b) FACS analyses of cell cycle of proliferating and quiescent eMSC after HS. (c) FACS analyses of cell viability of proliferating and quiescent eMSC after HS. (d) RT-PCR analysis of the p21 level in proliferating and quiescent cells. Data of three independent experiments are presented. (e, f) X-Gal staining. The progeny of HS-survived cells from proliferating (e) and quiescent (f) cultures. Ob: 10x; scale bar = 140 *μ*m.

Another feature of SIPS is a cell cycle arrest. Heat stress arrested the proliferation of both cell types while control cells maintained at 37°C were actively dividing (Figure 5(c)). At 24 h after HS, quiescent cells were stopped predominantly in the G0/G1 phase whereas proliferating cells were arrested both in the G0/G1and G2/M phases (Figure 5(b)). Although the total cell number in the G2/M and S phases was high (about 50%), this pattern of cell cycle distribution of proliferating cells remained unchanged during the following 72 h of recovery. Figure 5(c) demonstrates that these cells are not able to divide. Quiescent cells exposed to HS and returned to the normal temperature conditions resumed proliferation after 48 h of recovery. The pattern of their cell cycle distribution changed. Cell number in the G0/G1 phase decreased whereas in the G2/M and S phases, it increased from 18 to 34% during recovery for 72 h. Their cell cycle distribution became similar to unheated growing cultures (Figure 5(b)). Proliferative status of cells was also verified by Ki-67 expression.  Figure 5(a) demonstrates cells stained with antibodies to Ki-67. It is seen that at 37°C, there are a few Ki-67-positive cells in quiescent cultures whereas most cells are stained with these antibodies in proliferating cultures. After HS, the number of Ki-67-positive cells in cultures that returned for recovery under normal conditions was altered. It drastically declined in proliferating cultures exposed to HS and increased in heated quiescent cultures.

We also evaluated the expression of p21 protein, a cyclin-dependent kinase inhibitor.  Figure 5(d) demonstrates the different expression of p21 in quiescent and proliferating cultures before HS. It is higher in quiescent cells before HS (Figure 5(d)). At 24 h after HS, p21 expression increased in both cell types. In heated growing cells, high p21 level persisted for 72 h of recovery (Figure 5(d)) whereas in quiescent cells exposed to HS and returned under normal conditions, it drastically decreased and became lower than that in unheated quiescent cells after 72 h (Figure 5(d)). Collectively, the results presented in Figures 5(a)–5(d) show that during 72 h of recovery, quiescent cells resume proliferation whereas growing cells do not. Heated cells were harvested and subcultured. The progeny of HS-survived cells from both proliferating and quiescent cultures produce a monolayer (Figures 5(e) and 5(f)). Most cells are viable and X-Gal negative. Thus, HS-treated cells resumed proliferation but cycling cells did it more slowly.

### 3.6. Reactive Oxygen Species (ROS) and Antioxidative Defense in Quiescent and Proliferating Cells after HS

**Figure 6:**
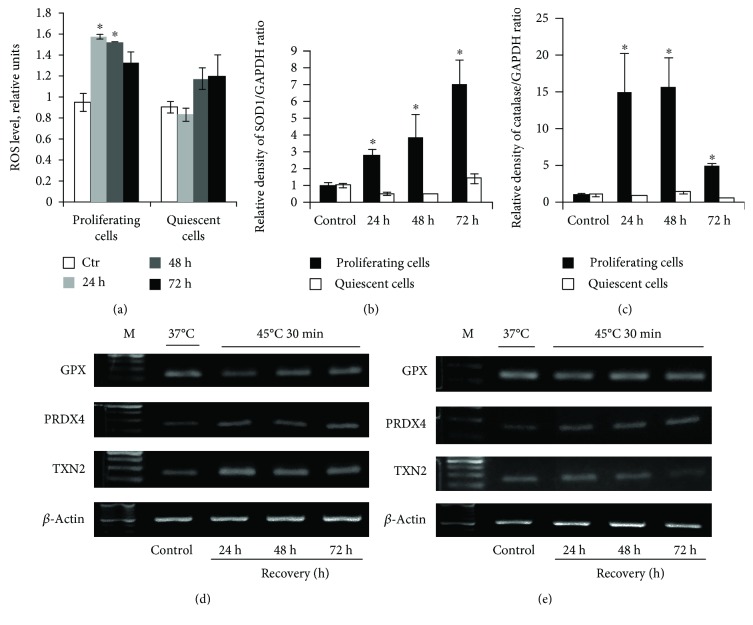
Oxidative defense in quiescent and proliferating cells after HS. (a) H_2_DCFDA-based FACS analysis of the ROS level in proliferating and quiescent cells. Analysis was performed 24, 48, and 72 h after treatment; all data were normalized to the control values. ^∗^The difference vs unheated cells at the day of treatment. One-way ANOVA with post hoc Tukey HSD test, *p* < 0.05. (b, c) qRT-PCR analysis of the SOD1 and catalase gene level in proliferating and quiescent cells. ^∗^The difference vs unheated cells (*t*-test, *p* < 0.05). (d) RT-PCR analysis of the GPX, PRDX4, and TXN2 levels in proliferating cells. (e) RT-PCR analysis of the GPX, PRDX4, and TXN2 gene levels in quiescent cells. Data of three independent experiments are presented.

It is known that hyperthermia increases ROS production.  Figure 6(a) demonstrates the ROS level in both cell types after HS. It is seen that ROS production was elevated in proliferating but not in quiescent cells exposed to HS. The increase in the ROS level may be accompanied by modified expression of genes involved in the antioxidant defense. We examined the expression of catalase, SOD1, PRDX4, GPX, and TXN2 genes controlling the synthesis of ROS-scavenging and other redox-active proteins. It was found that PRDX4 was enhanced in both cultures during 72 h after heating (Figures 6(d) and 6(e)). Expression of catalase, SOD1, and TXN2 genes was increased in heated cycling cells while it was unchanged in cells that underwent HS at the quiescent stage. The level of SOD1 caused by HS drastically increased during 72 h of recovery while catalase expression enhanced during 48 h was then dropped (Figures 6(b) and 6(c)). GPX expression in both cell types remained unaltered (Figures 6(d) and 6(e)). These results show HS-enforced ROS production in heated proliferating cells that was accompanied with increased expression of genes regulating ROS-scavenging and protein-reducing enzymes. In contrast, HS did not alter ROS production in quiescent cells and genes involved in antioxidant defense were mostly silent.

### 3.7. Differentiation and Transformation Potential of HS-Survivor Cells Assayed with NGS

**Figure 7:**
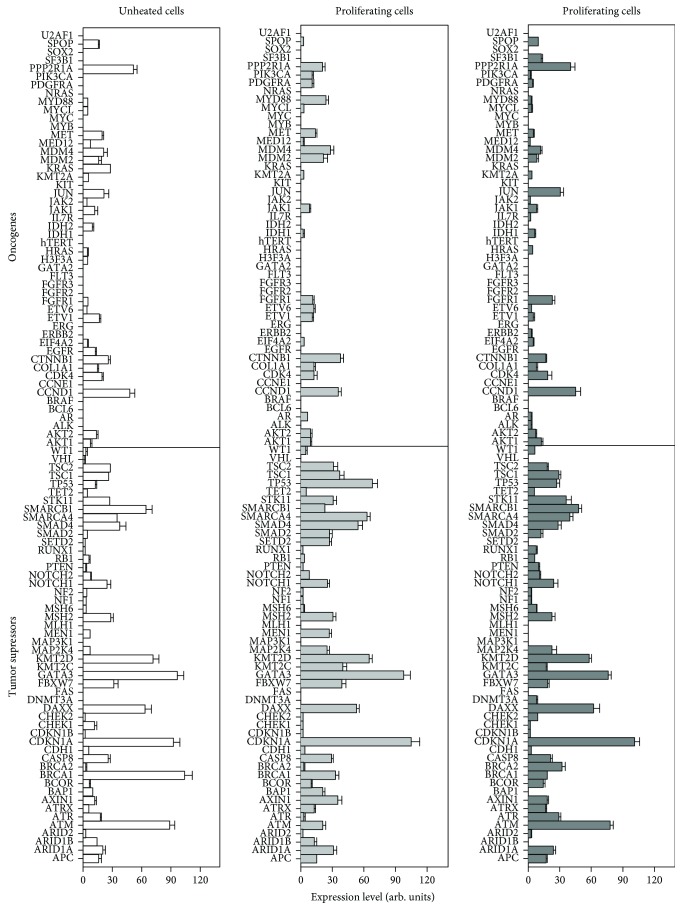
Tumor suppressor and oncogene expression in unheated and HS–survived eMSC progeny.

To investigate cell transformation potential, we evaluated expression of tumor suppressor genes (TSGs) and oncogenes (ONGs). The data presented in Figure 7 indicate that the general pattern of TSG and ONG expression remains similar for all cell types although some gene activity may vary. The average expression for ONGs in unheated cells and heated proliferating and quiescent cells comprised 7.19 ± 0.92, 6.87 ± 0.78, and 6.79 ± 0.68 arb. units. For TSGs, the corresponding means were 21.8 ± 1.78, 22.3 ± 1.69, and 22.7 ± 1.96. Thus, our data revealed no significant difference in TSG or ONG expression between studied cell types. The absence of tumorigenic potential in all cell types was also evident from no expression of several severe oncogenes, including Myc, hTERT, HRAS, NRAS, Pi3K, KIT, SOX2, BCL6, and FLT, and from the expression of all main TSGs including TP53, PTEN, STK11, TSC1, TSC2, CDKN1A, SMAD2, and SMAD4. Thus, our data indicate that the progeny of heated proliferating and quiescent cells as well as unheated cells maintain the safe TSG and ONG profile and do not exhibit transformation potential.

**Figure 8:**
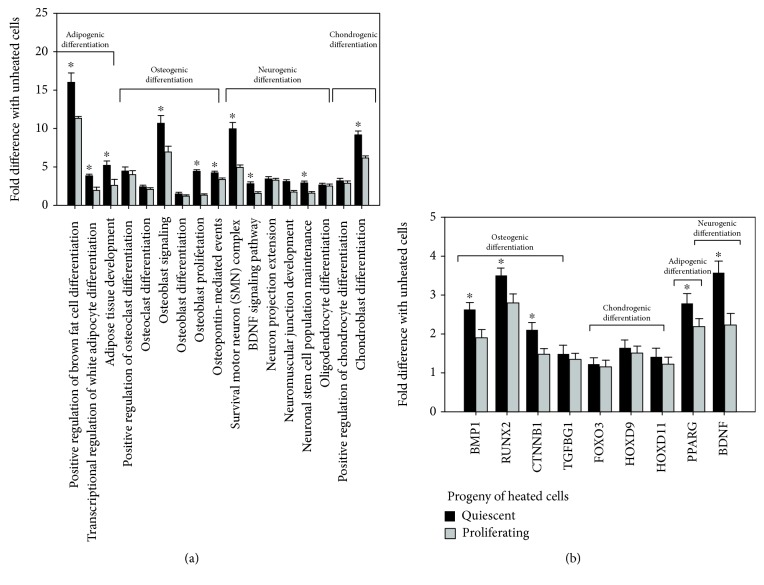
Differentiation potential of HS-survived eMSC progeny. (a) Gene modules enriched for differentiation-related genes; (b) expression of transcription factors (TFs) involved in MSC differentiation. ^∗^Statistically significant difference between HS-survived heated quiescent and proliferating cells (*p* < 0.01).

Cell differentiation potential was assessed by GO biological processes and BioSystems molecular pathways enriched for genes related to differentiation (Figure 8, Table S, supplement). We found (Figure 8(a)) that the progeny of HS-survived cells exhibited the induction of 15 modules related to the differentiation of mesodermal lineage (adipogenesis, osteogenesis, and chondrogenesis) and two modules implicated in the differentiation of ectodermal lineage (neurogenesis). Differentiation potential of survived quiescent cell progeny was higher than that of the progeny of heated cycling cell. To further verify the induction of gene modules related to differentiation, we examined the expression of transcription factors (TFs) driving osteogenic, adipogenic, chondrogenic, and neurogenic differentiation [32].  Figure 8(b) demonstrates that TFs involved in differentiation were expressed in progeny of heated cells. The level of their expression was higher in HS-survived quiescent cells.

## 4. Discussion

Cell response to stress factors is under intensive examination. Much of the current knowledge of cell stress responses is based on experiments with cycling cultured cells. Less is known about the effects of stress on cells in distinct phases of the cell cycle. Chinese hamster ovary cells of the middle and late S phases are more susceptible to stress than cells in mitosis, early S, G1, and G2 phases of the cell cycle [36]. HeLa cells and human skin fibroblasts were more sensitive to heat stress in the early than late S phase [37, 38]. The comet assay showed what the highest level of DNA damage in cells exposed to etoposide, a topoisomerase II inhibitor, was observed in G2 [39].

Stem cell response was also basically examined with proliferating cells in culture. In a living body, stem cells remain in the quiescent state for prolonged periods of time, entering the cell cycle in response to local signals of damage and other regeneration needs. Very few studies have explored stress response of MSC in the quiescent state. It has been demonstrated that quiescent MSC are more resistant to anoxia and metabolic stress than cycling cells [27]. Genome-wide transcriptional profiling of quiescent cells revealed a distinct shift in the transcription factor landscape in response to the restriction of energy supply [40].

In our experiments, we have modeled cellular quiescence to compare the stress response of the quiescent and proliferating mesenchymal stem cells. Experiments have been performed on eMSC from desquamated endometrium of menstrual blood. It is considered that these cells are derived from shed endometrium. The cells are multipotent, capable for self-renewal, express CD13, CD29, CD44, CD73, CD90, and CD105 markers, and are negative for the hematopoietic markers CD34 and CD45 [25]. The interest to these cells emerged from their origin. They are considered to be putative cells that participated in the regeneration and remodeling of the endometrium. The human endometrium is a highly dynamic tissue with the ability to regenerate each month during the woman reproductive life.

Quiescence is a reversible cell cycle arrest during which the cells reside in the G0/G1 phase. They can enter the proliferative stage in response to growth signals. Different approaches are applied to mimic MSC quiescence in culture [41]. Coating of the culture dish surface with hyaluronan holds most human placenta-derived MSC in the G0/G1 state compared to the cells grown on the standard tissue culture surface [42]. Earlier, the authors demonstrated that hyaluronan substratum induced multidrug resistance in these cells [43]. However, the quiescence state of cultured MSC is usually modeled by cell cultivation in serum-free conditions. It should be emphasized that MSC are sensitive to serum starvation and extended serum deprivation induced cell death [44]. We chose 30 h serum starvation that did not provoke detectable cell damage. Most cells were accumulated in the G0/G1 phase, were Ki-67 negative, and did not express cyclin A2 and its functional target pRb proteins involved in the transition from the G0/G1 to the S phase of the cell cycle [45].

These cells were subjected to HS. HS is one of the most conserved cellular stress responses. It is characterized by transcription and accumulation of heat shock proteins (HSP). Chaperones have been defined as proteins that bind to and stabilize an otherwise unstable conformer of another protein. With controlled binding and release of the substrate protein, they facilitate its correct fate. Among various HSPs, HSP70, with a molecular weight of 70 kDa, is known to be the major molecular chaperone. In unstressed cells, HSP70 exists in the cytoplasm. HS as well as other stressful factors exhibits robust enhancement of HSP70 expression and its relocation from the cytoplasm into the nuclei [46]. During recovery, HSP70 leaves the nuclei and becomes distributed throughout the cytoplasm. HSP expression returns to the level of intact cells. In our experiments, we monitored the expression of HSP70, a highly inducible molecular chaperone. We found that HS triggered the expression of HSP70 gene in both cell types. It was at the high level in proliferating cells up to 72 h of postthermal stress whereas it started to decline in quiescent-heated cultures after 48 h and dropped close to the level in unheated cells after 72 h cultivation under normal culture conditions. Immunofluorescence assessment showed localization of HSP70 protein in nuclei of both cell types at 2 h after HS. At 24 h after cell recovery, the pattern of HSP70 distribution was different in proliferating and quiescent cells. HSP70 was hardly identified in heated quiescent cells but visible in both the nucleus and cytoplasm of heated proliferating cells. These findings demonstrate that quiescent and proliferating cells displayed different HS responses. Cells heated in the quiescence state are more resistant to HS as they exhibit more rapid return of HSP70 expression and distribution to the norm than growing cells that underwent HS.

It is commonly believed that triggering of the senescence program with various stresses is initiated by DNA damage [34, 35]. Heat shock as a stressful factor induces both single- and double-strand DNA breaks. DNA breaks activate DNA damage response (DDR). In most somatic cells, DDR is initiated by activation of sensor kinases of ATM (ataxia telangiectasia mutated), ATR (ATM and Rad related), and DNA-PK (DNA-dependent protein kinase). They recognize DNA damage and phosphorylate downstream members of the signaling cascade, including histone H2AX [47]. Phosphorylated H2AX (*γ*H2AX) foci are required for recruiting and anchoring of DDR participants in sites of DNA damage. *γ*H2AX foci are easily detected by fluorescent microcopy that allows using them as a reliable DDR marker.

Quiescent and proliferative cells differed in DDR response elicited by HS. The cell heating was accompanied with generation of *γ*H2AX foci. Their maximal production was registered after 24 h recovery, and afterwards, it started to decline. After 72 h of cell cultivation under standard conditions, only few cells heated in quiescence had *γ*H2AX foci but the foci were still identified in cells exposed to HS in the proliferative phase. ATR (another DDR marker) is weakly expressed in intact cells of both types. Its expression was increased during 24 h in heated cycling cells and then dropped. In cells heated at the quiescence state, ATR expression was not activated by HS. The difference in ATR expression in two cell types is, probably, attributed to the different pattern of the cell cycle distribution in cultures subjected to HS. The difference is mostly concerned to the cell number in the S phase. In quiescent cells that underwent HS, 4% of the cells stayed at the S phase whereas HS-treated proliferating culture had 14% cells in the S phase. Most reports on ATR function link ATR to the important role during the S phase [48–50]. Culture with low cell number in the S phase is more resistant to damage, and ATR expression is not required for DNA damage repair.

In our recent experiments, HS induced preliminary senescence (SIPS) in eMSC. It was accompanied by modified cell morphology, cell cycle arrest, and enhanced expression of p21 protein, an inhibitor of cyclin-dependent kinases [25]. Now, we have shown that SIPS was induced in both quiescent and proliferating eMSC cultures after HS. Both cell types after HS stopped to proliferate, became negative for expression of proliferation marker Ki-67, acquired flattened, enlarged morphology, were positive for SA-*β*-Gal staining, and exhibited higher expression of p21 protein. Cells that survived HS gradually restored the normal phenotype under normal culture conditions. Quiescent-heated cells did it more rapidly than cycling cells. By 72 h after HS, most cells did not display SIPS features whereas cells exposed to HS in the proliferative state still retained SIPS phenotype.

ROS are well known to be implicated in various important cellular processes including signaling, regulation of homeostasis [51]. However, excessive ROS produce significant damages in the cell structures and functions affecting cell proliferation, causing genomic instability, cellular senescence, or death. Various kinds of stress including overheating lead to an overproduction of ROS [52].

In our experiments, HS enhanced the ROS level in cycling but not quiescent cells. In growing cells, HS evoked enhanced ROS production and expression of catalase and SOD1, PRDX4, GPX, and TXN2 genes. Supposedly, in quiescent cells, the system of the oxidative defense copes with the task to keep the ROS amount at the threshold level more rapidly than in growing cells. It is supported by unperturbed expression of catalase, SOD1, GPX, and TXN2 controlling the synthesis of redox-active proteins.

Spontaneous malignant transformation of MSC during prolonged in vitro expansion is of concern. It is currently believed that normal human MSC do not undergo spontaneous transformation in vitro during their passaging. Less is known about tumorigenic potency of stress that survived MSC. We performed mRNA sequencing and assessed expression of 50 TSGs and 49 ONGs [30] to examine tumorigenic potential of cultured HS-survived cells. All cells show no signs of immortalization. We revealed the silencing of all principal oncogenes and expression of key tumor suppressors in all investigated cell types (unheated control and cells subjected to HS in quiescent or proliferating stages). These results are in a good agreement with our previous results indicating that the progeny of heated human MSC did not show hallmarks of cancer [31]. Moreover, long-term cultivation of MSC that survived HS resulted in the replicative senescence [25].

The progeny of cells heated in the quiescent or proliferative stages retained the capacity for differentiation common for MSC. HS-survived cells heated in quiescence exhibited even higher differentiation potential compared to the progeny of cycling cell subjected to HS. Earlier, we reported that human eMSC expanded after HS were able to differentiate into adipocytes and osteoblasts as well as transdifferentiate into neuronal cells [25]. It was reported that the progeny of human MSC that underwent genotoxic stress or unfavorable conditions [53, 54] maintained differentiation potential. Thus, NGS data demonstrated that cells that endured stress retain the differentiation potency and show no signs of tumorigenic risk.

Collectively, we demonstrate the higher stress tolerance of quiescent human mesenchymal stem cells compared to cycling cells. In organism, stem cells face numerous challenges and should be protected from premature exhaustion to ensure tissue maintenance. It can be speculated that transplantation of cell accumulated in the quiescent state by serum starvation suggests a new, easy to implement, and safe strategy to improve transplanted cell survival and effectiveness of stem cell therapy.

## Figures and Tables

**Table 1:** The primers and conditions for qRT-PCR and Q-PCR.

Symbol	Primer sequence	Amplification conditions	PCR product size (bp)	Accession number
HSP70	F 5′ atgcggccaagaaccaggtg 3′ R 5′ gcgctgcgagtcgttgaagt 3′	93°C, 20 s, 61°C, 20 s, 72°C, 30 s	307	NM_005345.5
p21	F 5′ccacatggtcttcctctgctg 3′ R 5′ gatgtccgtcagaacccatg 3′	93°C, 20 s, 55°C, 20 s, 72°C, 30 s	316	NM_001220778.1
Actin	F 5′ gccgagcgggaaatcgtgcgt 3′ R 5′ cggtggacgatggaggggccg 3′	93°C, 20 s, 70°C, 20 s, 72°C, 30 s	506	NM_001101.3
FOD1	F 5′ ggtcctcactttaatcctctat 3′ R 5′catctttgtcagcagtcacatt 3′	93°C, 20 s, 55°C, 20 s, 72°C, 30 s	97	NM_000454.4
hCAT	F 5′ ttaatccattcgatctcacc 3′ R 5′ ggcggtgagtgtcaggatag 3′	93°C, 20 s, 57°C, 20 s, 72°C, 30 s	210	NM_001752.3
hGPX	F 5′ cgccaccgcgcttatgaccg 3′ R 5′ gcagcactgcaactgccaagcag 3′	93°C, 20 s, 66°C, 20 s, 72°C, 30 s	238	NM_001329455.1
TXN2	F 5′ ggtgatggccaaggtgga 3′ R 5′agggaggcagcaggaagg 3′	93°C, 20 s, 60°C, 20 s, 72°C, 30 s	257	XM_006724226.1
PRDX4	F 5′agcgccctactgggaagg 3′ R 5′ tggcccaagtcctccttg 3′	93°C, 20 s, 63°C, 20 s, 72°C, 30 s	262	XM_017029231.1
ATR	F 5′ agtgcctcgcagcctcag 3′ R 5′ctgcctttggcctcatgg 3′	93°C, 20 s, 63°C, 20 s, 72°C, 30 s	303	NM_001354579.1
GAPDH	F 5′ gactcatgaccacagtccatgc 3′ R 5′ agaggcagggatgatgttctg 3′	93°C, 20 s, 67°C, 20 s, 72°C, 30 s	113	NM_001289746.1

## Data Availability

The data used to support the findings of this study are available from the corresponding author upon request.

## Supplementary Materials

Supplementary Materials Table S: gene ontology^1^ biological processes and BioSystems^2^ molecular pathways that are enriched in genes with differential expression between unheated cells and progeny of heated quiescent and heated proliferating cells.
