# Supplementary material for: Quiescent Human Mesenchymal Stem Cells Are More Resistant to Heat Stress than Cycling Cells
Source: Stem Cells Int. 2018 Dec 24;2018:3753547. doi: 10.1155/2018/3753547 (PMC6323451; doi:10.1155/2018/3753547)
Supplement: Supplementary Materials — Table S: gene ontology1 biological processes and BioSystems2 molecular pathways that are enriched in genes with differential expression between unheated cells and progeny of heated quiescent and heated proliferating cells. [file 3753547.f1.doc]

**Table S. Gene Ontology ^1^ biological processes and BioSistems^2^ molecular pathways that are enriched in genes with differential expression between unheated cells and progeny of heated quiescent and heated proliferating cells.**

| **Pathway ID** | **Pathway name** | **Fold*** | **Gene number** | **p-value**** | **q-value**** | | **Pathway genes***** |
| --- | --- | --- | --- | --- | --- | --- | --- |
|  | **Progeny of heated quiescent cells** | | | | | |  |
| **GO:0090336** | **Positive regulation of brown fat cell differentiation** | **16.07±1.21** | **5** | **8.01E-03** | | **0.0505** | **MAPK14; MECOM; METRNL; INS; PRDM16** |
| **REACT_27161** | **Transcriptional regulation of white adipocyte differentiation** | **3.86±0.23** | **44** | **5.20E-03** | | **0.0588** | **SREBF2; CEBPD; RELA; HDAC3; EP300; NCOR1; MED8; MED19; TBL1X; MED9; MED10; PPARGC1A; MED31; SMARCD3; NFKB1; RXRA; MED13L; MED4; KLF5; NCOA6; NR2F2; CDK4; SREBF1; CREBBP; CEBPB; TGFB1; NCOR2; HELZ2; MED25; MED15; CARM1; MED1; MED27; MED23; MED30; MED24; ADIPOQ; SLC2A4; MED12; MED29; TBL1XR1; MED16; PPARA; NCOA** |
| **GO:0060612** | **Adipose tissue development** | **5.24±0.52** | **16** | **3.16E-03** | | **0.0562** | **ZNF516; CSF1; OXCT1; AACS; PPARD; ARRDC3; PIK3CA; ID2; ARID5B; XBP1; CDK4; TBL1XR1; DYRK1B; FTO; ATF2; LRP5** |
| **GO:0045672** | **Positive regulation of osteoclast differentiation** | **4.49±0.53** | **7** | **0.0254** | | **0.1302** | **CREB1; KLF10; CCR1; FOS; GNAS; TRAF6; ATP6AP1** |
| **KEGG__ hsa04071** | **Osteoclast differentiation** | **2.42±0.24** | **71** | **9.40E-03** | | **0.0852** | **FOSL2; NFKB2; TGFB2; RELA; MAPK3; ITGB3; MITF; MAP3K7; TYK2; ACP5; IFNGR2; PPP3CC; IKBKB; LILRB1; SOCS1; NFKB1; MAPK14; OSCAR; AKT2; CREB1; TGFBR1; SQSTM1; PPP3CA; AKT1; IKBKG; TNFRSF1A; GRB2; RELB; SOCS3; MAPK1; CYBA; PIK3CB; PIK3CG; JUNB; TGFBR2; STAT1; PIK3CA; JUND; FOS; PIK3R1; PIK3R2; TNFRSF11B; FYN; RAC1; AKT3; CTSK; TGFB1; STAT2; IFNAR1; JUN; FOSL1; MAP2K1; TRAF6; NFKBIA; FHL2; JAK1; SIRPA; PPP3CB; MAP2K7; PPP3R1; IFNAR2; TRAF2; MAPK13; IFNGR1; IRF9; PIK3CD; LILRA2; SYK; CSF1R; FCGR3A; MAPK9** |
| **WP322** | **Osteoblast signaling** | **10.69±1.05** | **8** | **0.0401** | | **0.1323** | **ITGB3; PDGFB; PDGFRA; COL1A1; PDGFRB; TNFRSF11B; PTH1R** |
| **GO:0001649** | **Osteoblast differentiation** | **1.52±0.27** | **68** | **0.0497** | | **0.0865** | **TP53INP2; DHX9; GTPBP4; PTH1R; FBL; TNC; SEMA7A; FGF9; MYBBP1A; LGR4; RRAS2; MEN1; GLI1; SNRNP200; PHB; VCAN; BMP6; FASN; SYNCRIP; H3F3B; CYR61; HNRNPC; GPNMB; MRC2; SND1; JUNB; ADAR; FHL2; DDX21; ITGA11; MEF2D; CLTC; RPS11; IGFBP3; ATP5F1B; HNRNPU; JUND; COL1A1; TPM4; RSL1D1; CREB3L1; IGFBP5; ALYREF; BCAP29; RACK1; EPHA2; CAT; RPS15; COL6A1; SNAI2; WWTR1; LIMD1; RDH14; TMEM119; SMAD5; RRBP1; SMAD3; IARS; TNN; MSX2; SMAD1; LRP5; IFT80; DNAJC13** |
| **GO:0033687** | **Osteoblast proliferation** | **4.42±0.23** | **22** | **0.013** | | **0.1205** | **ID3; NBR1; HAND2; SEMA4D; TWIST2; RIOX1; MEN1; ID2; ID1; TRPM4; SUFU; NOTCH1; SKI; IGFBP5; HDAC7; LIMD1; TWSG1; CDK6; SMAD3; TOB1** |
| **137960__PID** | **Osteopontin-mediated events** | **4.24±0.26** | **23** | **0.0184** | | **0.1443** | **RELA; MAPK3; ITGB3; PIP5K1A; NFKB1; ROCK2; MAPK1; MMP2; PIK3CA; FOS; PIK3R1; BCAR1; PAK1; GSN; RAC1; CD44; JUN; RHOA; NFKBIA; ILK; CDC42; SYK; PLAU** |
| **KEGG__M00426** | **Survival motor neuron (SMN) complex** | **9.99±0.84** | **6** | **0.0487** | | **0.1222** | **GEMIN4; GEMIN6 ;STRAP; DDX20; GEMIN7; GEMIN5; GEMIN4** |
| **WP2380** | **BDNF signaling pathway** | **2.87±0.26** | **87** | **0.0268** | | **0.0833** | **EIF2S1; RELA; MAPK3; SH2B1; MEF2A; CYFIP1; IKBKB; RAF1; PLCG1; PTPRF; NFKB1; MAPK14; SRC; NFATC4; STAT5A; NSF; JAK2; CREB1; CDK5R1; DLG1; SQSTM1; TSC2; EIF4EBP1; AKT1; RPS6KA1; IKBKG; SH2B2; EIF4E; GRB2; CDK5; MAPK1; EIF2S2; BMP2; GSK3B; STAT1; PPP2CA; FOS; PIK3R1; CFL1; RPS6; PIK3R2; SHC1; FYN; CTNNB1; RAC1; VAV2; EEF2; SORT1; ELK1; FRS2; NCK2; RHOG; JUN; MAP2K2; MTOR; HRAS; MAP2K1; TRAF6; YBX1; RAP1A; NFKBIA; BAD; PRKCD; GNB2L1; DPYSL2; KCNN2; SIRPA; CDH2; CDC42; PRKAA1; CDKL5; EGR1; STAT3; MAP3K2; RPS6KB1; MARCKS; IRS1; APC; RANBP9; GRIA1; FOXO3; KSR1; BCL2L11; ACACB; PDPK1; CSNK2A1; MAPK9** |
| **GO:1990138** | **Neuron projection extension** | **3.43±0.21** | **31** | **0.0121** | | **0.1325** | **ULK1; STK11; SLC9A6; CYFIP1; AURKA; FLRT1; CPNE1; ALCAM; MAP1B; LAMB2; IQGAP1; POSTN; CYFIP2; CDK5; RAPH1; IMPACT; SEMA7A; USP9X; VCL; ITGB1; ULK2; DVL1; DDR1; KDM1A; PPP3CB; NRP1; PLXNA3; NRP2; NDEL1; PLXNA1; SEMA3A** |
| **GO:0007528** | **Neuromuscular junction development** | **3.10±0.32** | **23** | **0.307** | | **0.1471** | **NRDC; RER1; APP; ZC4H2; TNC; CACNB3; PDZRN3; ETV5; ERBB2; LAMB2; CACNB4; ANK3; KALRN; AFG3L2; F2R; DVL1; COL4A1; CACNB2; UNC13B; COL4A5; UTRN; UNC13A; LRRK2** |
| **GO:0097150** | **Neuronal stem cell population maintenance** | **2.96±0.28** | **10** | **0.0467** | | **0.1347** | **SRRT; ASPM; JAG1; MMP24; PCM1; SS18; CDH2; HOOK3; FOXO3; NOTCH1** |
| **GO:0048709** | **Oligodendrocyte differentiation** | **2.68±0.26** | **33** | **0.0366** | | **0.1494** | **LPAR1; LYN; EIF2B4; SRSF1; DUSP10; ERCC2; HDAC11; VTN; WASF3; SLC8A3; ERBB2; CLU; CDK5; OLIG2; EIF2B3; CNP; ID2; EIF2B2; CSK; GSTP1; GSN; BNIP3; CD9; TGFB1; EIF2B5; ID4; BOK; MED12; CDKN2C; NKX6-2; SOX11; OLIG1; NOTCH1** |
|  | **Progeny of heated proliferating cells** | | | | | |  |
| **GO:0090336** | **Positive regulation of brown fat cell differentiation** | **11.37±0.21** | **5** | **0.0266** | | **0.0902** | **MAPK14; MECOM; METRNL; INS; PRDM16** |
| **REACT_27161** | **Transcriptional regulation of white adipocyte differentiation** | **1.96±0.21** | **44** | **4.32E-02** | | **0.1205** | **THRAP3; SREBF2; CCND3; CEBPD; MED22; CREBBP; RELA; NFKB1; NCOA3; MED21; KLF5; MED8; NCOA6; TBL1X; MED28; PPARG; MED10; NCOR2; CDK4; MED19; NR2F2; CEBPB; MED7; SREBF1; MED25; MED15; TBL1XR1; FAM120B; TGFB1; CARM1; MED24; PPARA; MED12; MED1; HELZ2; SLC2A4; MED29; ZNF638; ADIPOQ; NCOA2; MED16; MED23; MED30; MED27** |
| **GO:0060612** | **Adipose tissue development** | **2.59±0.26** | **19** | **0.0158** | | **0.0621** | **SLC25A25; ACAT1; ZNF516; PPARD; PPARGC1A; EBF2; BBS4; ARID5B; XBP1; AACS; CDK4; PIK3CA; ID2; SPG20; FTO; TBL1XR1; DYRK1B; ATF2; LRP5** |
| **GO:0045672** | **Positive regulation of osteoclast differentiation** | **4.05±0.42** | **8** | **2.51E-03** | | **0.0622** | **CSF1; CREB1; KLF10; POU4F1; FOS; GNAS; ATP6AP1; TRAF6** |
| **KEGG__ hsa04071** | **Osteoclast differentiation** | **2.08±0.19** | **65** | **0.024** | | **0.1341** | **FOSL2; NFKB2; TGFB2; RELA; MAPK3; ITGB3; MITF; MAP3K7; TYK2; ACP5; IFNGR2; PPP3CC; IKBKB; LILRB1; SOCS1; NFKB1; MAPK14; OSCAR; AKT2; CREB1; TGFBR1; SQSTM1; PPP3CA; AKT1; IKBKG; TNFRSF1A; GRB2; RELB; SOCS3; MAPK1; CYBA; PIK3CB; PIK3CG; JUNB; TGFBR2; STAT1; PIK3CA; JUND; FOS; PIK3R1; PIK3R2; TNFRSF11B; FYN; RAC1; AKT3; CTSK; TGFB1; STAT2; IFNAR1; JUN; FOSL1; MAP2K1; TRAF6; NFKBIA; FHL2; JAK1; SIRPA; PPP3CB; MAP2K7; PPP3R1; IFNAR2; TRAF2; MAPK13; IFNGR1; IRF9; PIK3CD; LILRA2; SYK; CSF1R; FCGR3A; MAPK9** |
| **WP322** | **Osteoblast Signaling** | **6.91±0.78** | **7** | **0.025** | | **0.1341** | **ITGB3; ITGAV; PDGFRA; PDGFB; PDGFRB; COL1A1; TNFRSF11B** |
| **GO:0001649** | **Osteoblast differentiation** | **1.16±0.18** | **64** | **0.0426** | | **0.128** | **DHX9; RBMX; TNC; RRAS2; TP53INP2; FBL; ASF1A; VCAN; AKT1; HOXA2; HDAC4; SNRNP200; PHB; RUNX2; HSD17B4; SND1; WNT3A; HNRNPC; SMAD3; HSPE1; SYNCRIP; CYR61; GLI2; BMP2; JUNB; TWIST1; IARS; JUND; CREB3L1; ADAR; H3F3B; HNRNPU; MRC2; COL1A1; GPNMB; IGFBP3; TPM4; FASN; SEMA7A; RRBP1; ATP5B; RPS11; CLTC; RPS15; RSL1D1; BCAP29; IGFBP5; GNB2L1; FHL2; ALYREF; COL6A1; SMAD5; WWTR1; TMEM119; EPHA2; DDX21; RDH14; ITGA11; LIMD1; SNAI2; MEF2D; TNN; MSX2; SMAD1; CAT; LRP5; IFT80; DNAJC13** |
| **GO:0033687** | **Osteoblast proliferation** | **1.31±0.16** | **22** | **0.047** | | **0.138** | **ID3; NBR1; HAND2; SEMA4D; TWIST2; RIOX1; MEN1; ID2; ID1; TRPM4; SUFU; NOTCH1; SKI; IGFBP5; HDAC7; LIMD1; TWSG1; CDK6; SMAD3; TOB1** |
| **137960__PID** | **Osteopontin-mediated events** | **3.38±0.27** | **25** | **0.038** | | **0.136** | **RELA; MAPK3; ITGB3; PIP5K1A; NFKB1; ROCK2; MAPK1; MMP2; PIK3CA; FOS; PIK3R1; BCAR1; PAK1; GSN; RAC1; CD44; JUN; RHOA; NFKBIA; ILK; CDC42; SYK; PLAU** |
| **KEGG__M00426** | **Survival motor neuron (SMN) complex** | **4.95±0.43** | **6** | **0.015** | | **0.139** | **DDX20; GEMIN7; GEMIN5; GEMIN4; GEMIN6 ;STRAP** |
| **WP2380** | **BDNF signaling pathway** | **1.56±0.21** | **49** | **0.066** | | **0.183** | **TSC2; EIF4E; SH2B1; MAPK3; CYFIP1; CREB1; RAF1; STAT5A; CASP3; GRIP1; SHC3; EIF2S1; RELA; NFKB1; CDK5; BDNF; RPS6KA5; CHUK; CAMK4; GRB2; NFATC4; EIF4EBP1; NCF2; MAPK1; SQSTM1; PTK2B; PPP2CA; CRTC1; PIK3R2; PIK3R1; RAC1; CFL1; RHOG; IKBKG; CTNNB1; VAV2; SORT1; RAP1A; EEF2; STAT1; ELK1; EIF2S2; RPS6; FOS; FOXO3; BAD; YBX1; SIRPA; MAP2K2; SHC1; MARCKS; FRS2; TRAF6; EGR1; RACK1; CDH2; JUN; CDC42; PRKCD; MTOR; STAT3; RPS6KB1; DPYSL2; RANBP9; HRAS; MAPK9; GRIA1; APC; IRS1; KSR1; KCNN2; BCL2L11; PDPK1; MAP3K2; MAP2K1; CSNK2A1; ACACB; NFKBIA; PRKAA1** |
| **GO:1990138** | **Neuron projection extension** | **3.28±0.27** | **30** | **0.016** | | **0.134** | **CYFIP1; IMPACT; CPNE1; SLIT1; SEMA7A; CDK5; AURKA; STK11; IQGAP1; LLPH; RAPH1; EIF2AK4; POSTN; ALCAM; PLXNB1; LAMB2; USP9X; PLXNA1; ULK2; VCL; ITGB1; NDEL1; PPP3CB; SEMA3A; KDM1A; DDR1; NRP1; DVL1; PLXNA3; CYFIP2** |
| **GO:0007528** | **Neuromuscular junction development** | **1.73±0.18** | **22** | **0.021** | | **0.141** | **APP; NRDC; RER1; ALS2; FNTA; TNC; COLQ; PDZRN3; NEDD4; LAMB2; LRP4; DNAJA3; KALRN; UTRN; LRRK2; COL4A1; F2R; DVL1; AFG3L2; CACNB2; UNC13B; COL4A5** |
| **GO:0097150** | **Neuronal stem cell population maintenance** | **1.58±0.14** | **9** | **0.029** | | **0.131** | **PRRX1; SRRT; MMP24; PCM1; FOXO3; NOTCH1; SS18; CDH2; HOOK3** |
| **GO:0048709** | **Oligodendrocyte differentiation** | **2.48±0.26** | **7** | **0.044** | | **0.094** | **RHEB; PRMT5; PPARG; HDAC2; DAG1; MTOR; CXCR4** |

**1-Gene ontoloigy database** www.geneontology.org/

**2- Biosystems database** https://www.ncbi.nlm.nih.gov/biosystems/

***Expression fold in relation to unheated cells**

****The lowest significance for three replicates**

*****Genes are listed in descending order in regard to fold**
